# Comparison of tear proteome in allergic rhinoconjunctivitis patients and controls with respect to pollen season

**DOI:** 10.1111/all.13444

**Published:** 2018-04-15

**Authors:** P. V. Tomazic, L. Liesinger, B. Pucher, G. G. Thallinger, A. Leitner, S. Spoerk, C. Gerstenberger, D. Lang‐Loidolt, R. Birner‐Gruenberger

**Affiliations:** ^1^ ENT‐University Hospital Medical University of Graz Graz Austria; ^2^ Institute of Pathology Research Unit Functional Proteomics and Metabolic Pathways Medical University of Graz Graz Austria; ^3^ The Omics Center Graz BioTechMed‐Graz Graz Austria; ^4^ Institute of Computational Biotechnology Graz University of Technology Graz Austria; ^5^ Center of Medical Research Mass Spectrometry Core Facility Medical University of Graz Graz Austria; ^6^ The Austrian Center of Industrial Biotechnology Graz Austria; ^7^ Gottfried Schatz Research Center Molecular Biology and Biochemistry Medical University of Graz Graz Austria

To the Editor,

Tear fluid comprises electrolytes, lipids, mucins, and proteins among other components.^1^ Tear composition reflects the physiological condition of underlying tissue but can be altered in disease related to the eye or other organs and systemic diseases such as diabetes.^2^ Several tear proteomic studies for diseases other than allergic rhinoconjunctivitis exist proofing the feasibility of proteomic approaches to tear fluid.^3^, ^4^, ^5^, ^6^

Twenty‐one individuals (8 male, 13 female) with a mean age of 33 years (SD: 8.3 years) were prospectively included in the study group comprising 10 (48%) patients with allergic rhinoconjunctivitis (AR) and 11 (52%) healthy controls (HC). From patients with AR, tear fluid was collected in pollen season according to their sensitization pattern and the airborne pollen exposure (pollen counts) and symptoms present. On the same day, the same number of healthy controls was recruited from the outpatient department at the same time to have the same natural pollen exposure. The same patients and controls were recalled on the same day out of season. The day depended on the respective patients with AR. They must not have had symptoms, and natural pollen exposure must have been diminished during the out of season visit. Tear fluid was collected and sent to LC‐MS/MS mass spectrometry. For detailed methods, please see Data S1.

In total, 90 proteins could be identified and quantified in tear fluids. Given the requirement that the protein was filtered for 70% valid values of samples in at least one group, 22 different proteins were further analyzed (Table S1, online). Comparing seasonal and group differences, six proteins showed significantly different abundances (Figure 1).

**Figure 1:**
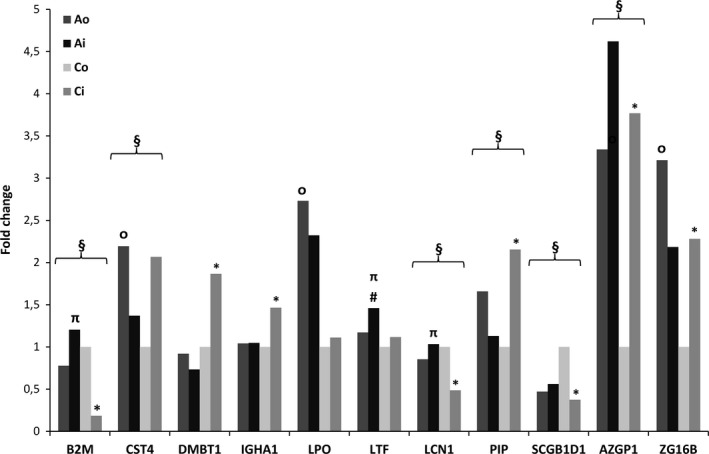
Significantly altered tear fluid proteins in allergic rhinoconjunctivitis in and out of pollen season. Reported are fold changes compared to controls out of season (Co). Significance is marked in allergic rhinoconjunctivitis (AR) out of season (Ao) vs in season (Ai) by #; in controls, out of season (Co) vs in season (Ci) by *; Ai vs Ci by π; Ao vs Co by ᴑ; and in 2‐way ANOVA by §; *P* < .05

Lactotransferrin was the only protein that was significantly less abundant (1.2‐fold) out of season in patients with allergic rhinoconjunctivitis than in season (Figure 1).

In contrast, in healthy controls, eight proteins were significantly altered in abundance out of season as compared to in season (Figure 1). Three proteins were found to be more abundant in HC out of season. On the other hand, five proteins had decreased abundance out of season.

Out of season, four different proteins were significantly more abundant in AR than in HC. In season, three proteins were significantly more abundant in AR than in HC (Figure 1).

The majority of pathways over both groups and seasons were related to the immune system (Figure S1). Others were transport of small molecules, vesicle‐mediated transport, metabolism of proteins, disease, and hemostasis.

Here, we investigated the tear fluid proteome of patients with allergic rhinoconjunctivitis and controls following them in and out of pollen season.

A major constituent of tear fluid is proteins that fulfill various functions.^1^ For the tear fluid proteome, up to around 1500 proteins have been identified in deep proteomic studies.^7^ Leonardi et al^5^ found 78 different proteins in vernal keratoconjunctivitis by iTRAQ quantitative proteomics which is a similar number of proteins found in the present study. Souza et al^4^ identified a large number of protease and protease inhibitors in tear fluid of one healthy subjects which was similar to findings we made in nasal mucus proteome. Soria et al^3^ analyzed tear proteome comparing dry eye and patients with meibomian gland disorder to healthy controls. They found 26 differently abundant proteins among which cystatin‐S was a candidate protein that also was significantly elevated in AR compared to HC in the present study. Yenihayat et al^6^ analyzed tear proteome in keratoconus. Among others, lipocalin was upregulated in patients, which was also significantly upregulated in patients with AR in season in our group.

Beta‐2 microglobulin/B2M is involved in the presentation of antigens to the immune system via the major histocompatibility I complex (MHC I), and elevated levels reflect an activated immune response.^8^ A defect of this protein can lead to severe upper respiratory tract infection.^9^ It was found to be much more abundant (sixfold) in AR than in HC showing an activated immune response in season. In HC, however, it was significantly less abundant in season compared to out of season (fivefold) which may suggest the immune response is downregulated in season despite natural pollen exposure. Another possibility is that the abundance changes result from a higher vascular permeability and that there is no active regulation of B2M.

Cystatin‐S/CST4 was significantly elevated in AR compared to HC out of season. CST4 is a cysteine protease inhibitor. Ghafouri et al^10^ found that cystatin‐S levels were lower in AR in season in nasal lavage fluid. We had similar findings in tear fluid and nasal mucus proteomes; however, the data did not reach statistical significance.^11^ CST4 may play an important role in AR as it appears to be reversely regulated in the tear proteome of AR as compared to HC in vs out of season.

Lactoperoxidase/LPO was also significantly more abundant in AR compared to HC out of season. LPO belongs to the innate immune system and serves as antimicrobial defense. Lactotransferrin/LTF also belongs to the innate immune system and was the only protein to be significantly more abundant in AR in season vs. out of season and was as well significantly elevated in AR in season compared to HC. These findings underline a perennial activation of the innate immune system of AR in tear fluid being accentuated during the pollen season.^12^

Lipocalin‐1/LCN1 was found to be significantly more abundant in AR compared to HC in season and was significantly less abundant in HC in season compared to out of season. Lipocalins have been described for various functions in tear fluid including tear viscosity but were thus far not brought into context with allergic rhinoconjunctivitis, but with keratoconus.^6^, ^13^ Their reduced abundance in season could be a reaction toward pollen stress in HC lowering tear viscosity enhancing clearing of allergens from the conjunctiva which was not observed in AR.

Zinc‐alpha‐2‐glycoprotein/AZGP1 and zymogen granule protein 16 homolog B/ZG16B were both significantly upregulated in AR out of season compared to HC and in season in HC compared to out of season. AZGP1 has similarity to MHC class I antigen‐presenting molecules and may have a role in immune response in tear fluid.^14^ ZG16B was significantly elevated in reflex tears.^15^ For HC, this may mean elevated clearance of pollen during pollen season similar to the effect of lipocalin.

In Summary, tear fluid protein shows a perennial inflammatory state in patients with allergic rhinoconjunctivitis accentuated in season and less plasticity and protective properties to the conjunctiva in season compared to healthy controls. In contrary, healthy controls show anti‐inflammatory proteins as well as proteins enhancing epithelial barrier and clearance of tear fluid, and hence, potentially, allergens significantly upregulated in season.

## FUNDING INFORMATION

The study was funded by the Austrian Science Fund (FWF) [Project Number: KLI 425 Programm Klinische Forschung (KLIF)].

## AUTHOR CONTRIBUTIONS

Peter Valentin Tomazic, M.D., Ph.D., designed the study, collected the data, prepared the manuscript, and performed statistical analysis. Laura Liesinger performed statistical analysis and proteomic analysis. Bettina Pucher performed statistical analysis and critically reviewed the article. Gerhard G. Thallinger performed statistical analysis and critically reviewed the article. Anita Leitner collected the data and prepared the sample. Stefan Spoerk performed proteomic analysis. Claus Gerstenberger contributed to graphical design and revision. Doris Lang‐Loidolt, M.D., designed the study, collected the data, critically reviewed the article, and performed statistical analysis. Ruth Birner‐Gruenberger, Ph.D., designed the study, performed proteomic analysis, prepared the manuscript, and performed statistical analysis.

## CONFLICTS OF INTEREST

The authors declare that they have no conflicts of interest.
