# Supplementary figures and images for: Comparison of tear proteome in allergic rhinoconjunctivitis patients and controls with respect to pollen season
Source: Allergy. 2018 Apr 15;73(7):1541–3. doi: 10.1111/all.13444 (PMC6033167; doi:10.1111/all.13444)

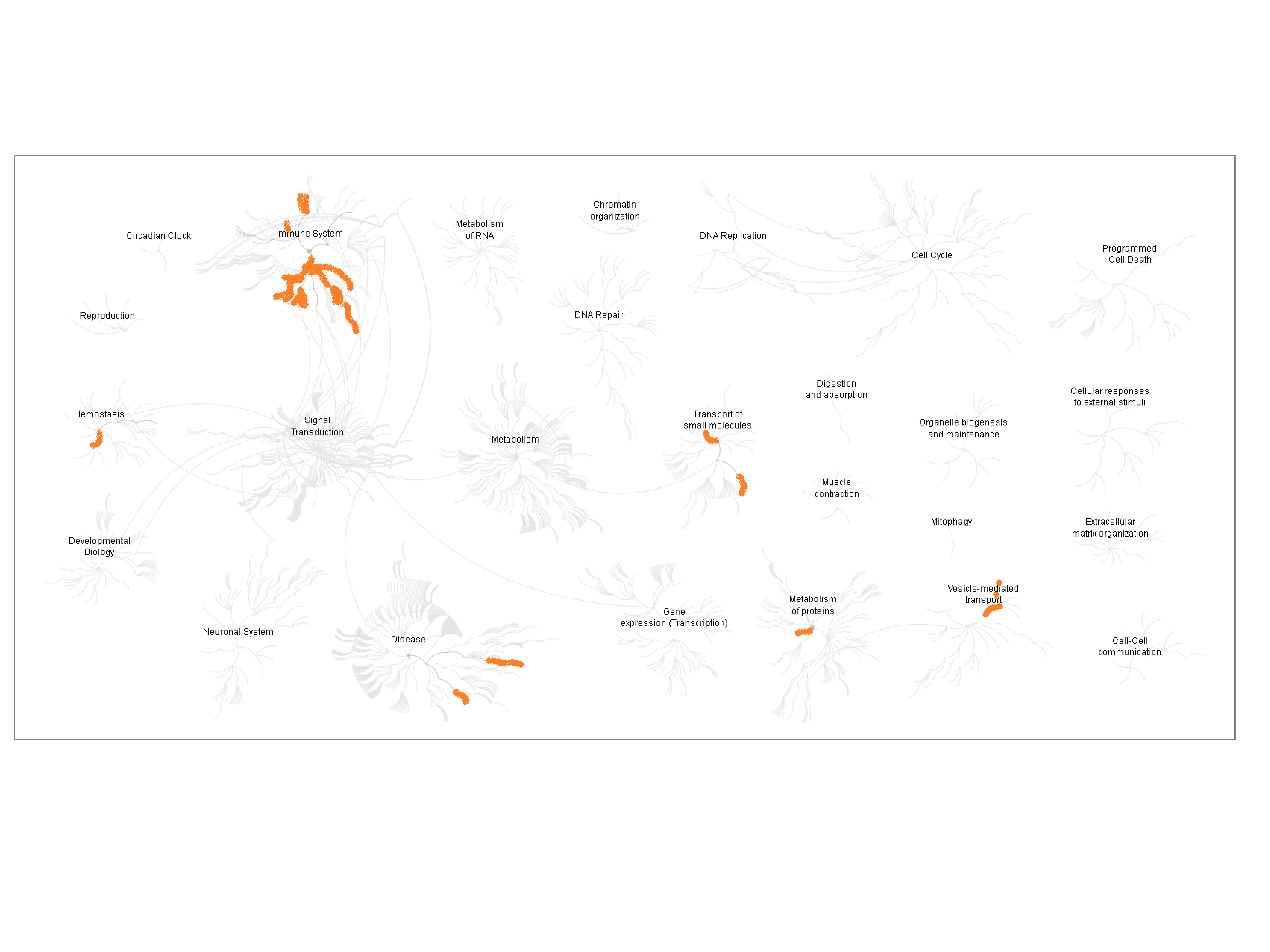

Supplement: Supplementary file 1 [file ALL-73-1541-s001.tif]
