# Supplementary material for: Comparison of tear proteome in allergic rhinoconjunctivitis patients and controls with respect to pollen season
Source: Allergy. 2018 Apr 15;73(7):1541–3. doi: 10.1111/all.13444 (PMC6033167; doi:10.1111/all.13444)
Supplement: Supplementary file 3 [file ALL-73-1541-s003.docx]

**Abbreviations**

DTT, dithiothreitol

FDR, false discovery rate

IAA, iodoacetamide

LC-MS/MS, liquid chromatography-tandem mass spectrometry

nano-HPLC, nano flow-high performance liquid chromatography

SD, standard deviation

SPT, skin prick test

**Material and Methods**

*Patients***:** Twenty-one individuals (8 male, 13 female) with a mean age of 33 years (SD: 8.3 years) were prospectively included in the study group comprising 10 (48%) allergic rhinoconjunctivitis (AR) patients and 11 (52%) healthy controls (HC). Allergy status was verified by patients history for symptoms of allergic rhinoconjunctivitis, skin prick tests (SPT, Allergopharma GmbH & Co. KG, Reinbek, Germany) and specific IgE (ImmunoCAP, Thermo Fisher Scientific Inc., Vienna, Austria) in all patients and controls. Patients sensitized to house dust mite or animals solely were excluded to avoid bias due to small sample size (Table 1). Thus, only patients sensitized to pollen and also showing rhinitis and conjunctivitis symptoms during the pollen season were considered for evaluation. Patients with acute and/or chronic sinusitis as defined by the EPOS [9] guidelines were also excluded, as were patients with malignant tumors and any infectious or cardiopulmonary disease, or who had been treated with systemic or topical drugs including antihistamines, corticosteroids, antibiotics, antifungals or any other immunomodulatory drugs in the four weeks prior to the study. The same exclusion criteria applied to the controls, who were healthy volunteers. Informed consent was obtained from all participants (allergics and controls) before enrolment. The study was approved by the institutional review board of the Medical University of Graz (approval number: .20-045 ex 08/09) and registered at ClinicalTrials.gov (Identifier: NCT02159404)

*Sample collection:* In pollen season (i) (with clinical symptoms present in allergic rhinoconjunctivitis patients) and out of pollen season (o) (without clinical symptoms in allergic rhinitis patients) tear fluid was collected with the glass capillary method [10]. Healthy controls’ samples were collected on the same day as allergic rhinitis patients’ samples. Tear fluid volume obtained was equal in both groups. Then, tear fluid was deep-frozen at -92° Celsius before processing for LC-MS/MS mass spectrometry.

*Sample preparation:* After defrosting samples were mixed and centrifuged at 13000 rpm for 5 min to remove insoluble particles. Protein content was estimated by Bradford assay (Bio-Rad, Vienna, Austria). 50 µg of protein was solubilized to 35 µl of 100 mM ammonium hydrogen carbonate, reduced with 35 µl of 10 mM DTT and alkylated with 8 µl of 55 mM IAA. Then samples were homogenised in ultrasound bath (10 minutes) with an additional 78 µl of 100 mM ammonium hydrogen carbonate. Protein was digested with 1 µg modified trypsin (Promega, Vienna, Austria) by shaking at 550 rpm over night at 37°C. Samples were acidified with 3 µl 5 % formic acid. Completion of digest was controlled by 4-12% SDS-PAGE of 2 µg aliquots of digested versus undigested samples and silver staining.

*Mass spectrometric analysis:* 40 µl, i.e. 2 µg, were separated by nano-HPLC on an Agilent (Vienna, Austria) 1200 system equipped with a Zorbax 300SB-C18, 5µm, 5 x 0.3mm enrichment column and a Zorbax 300SB-C18, 3.5 µm, 150 x 0.075 mm nanocolumn. Samples were injected and concentrated on the enrichment column for 6 min using 0.1 % formic acid as isocratic solvent at a flow rate of 20 µL/min. The column was then switched into the nanoflow circuit, and the sample was loaded on the nanocolumn at a flow rate of 300 nL/min and separated using the following gradient: solvent A: water, 0.1% formic acid; solvent B: acetonitril/water 80/20, 0.1% formic acid; 0-10 min: 10% B; 10-130 min 10-60% B, 130-132 min 60-95% B, 132-140 min 95% B, 140-140.01 min 95-10%. The sample was ionized in the nanospray source equipped with nanospray tips (PicoTipTM Stock# FS360-75-15-D-20, Coating: 1P-4P, 15+/- 1µm Emitter, New Objective) and analyzed in a Thermo Scientific (Vienna, Austria) LTQ-FT mass spectrometer in positive ion mode by alternating full scan MS (m/z 400 to 2000) in the ICR cell and MS/MS by CID of the 5 most intense peaks in the ion trap with dynamic exclusion enabled (for a duration of 10s).

*Mass spectrometric data analysis:* The LC-MS/MS data were analysed by MaxQuant by searching the public Swissprot database with taxonomy homo sapiens (downloaded on 02.03.2017, 20233 sequences) and common contaminants. Carbamidomethylation on Cystatin was entered as fixed modification, oxidation on methionine as variable modification. Detailed search criteria were used as follows: trypsin, max. missed cleavage sites: 2; search mode: MS/MS ion search with decoy database search included; precursor mass tolerance +/- 4.5 ppm; product mass tolerance +/- 20 ppm; acceptance parameters for identification: 1 % PSM FDR; 1 % protein FDR. In addition a label free quantitation was performed using MaxQuant [11] requiring a minimum of 2 ratio counts of quantified razor and unique peptides. Proteins were further annotated using Uniprot data ([www.uniprot.org](http://www.uniprot.org)) and NEIbank (www.neibank.nei.nih.gov).

*Statistics:* For statistical analysis of protein concentrations a Mann Whitney-U test was performed for group differences and a Wilcoxon test was performed for seasonal differences with a p-value of <0.05 considered significant.

For statistical analysis of proteomic data Perseus [12] was used to filter data for reverse and contaminants and at least 70% valid values in at least one group. Data were further on log2 transformed and missing values imputed from normal distribution (total matrix). A paired t-test (AR i vs. AR o, HC i vs. HC o), Welch’s t-test (AR i vs. HC i, AR o vs. HC o) and 2-way ANOVA (AR vs. HC, i vs. o) was employed to identify significantly altered proteins, respectively. Multiple testing corrections were performed with R by the Benjamini-Hochberg method.

The mass spectrometry proteomics data was deposited to the ProteomeXchange Consortium [13] (<http://proteomecentral.proteomexchange.org>) via the PRIDE partner repository with the data set identifier PXD 006533. (only for peer review: Reviewer account details: Username: [reviewer71102@ebi.ac.uk](mailto:reviewer71102@ebi.ac.uk); Password: AZXpTGRh)
